# Real-Time Integration of Optical Coherence Tomography Thickness Map Overlays for Enhanced Visualization in Epiretinal Membrane Surgery: A Pilot Study

**DOI:** 10.3390/bioengineering12030271

**Published:** 2025-03-10

**Authors:** Ferhat Turgut, Keisuke Ueda, Amr Saad, Tahm Spitznagel, Luca von Felten, Takashi Matsumoto, Rui Santos, Marc D. de Smet, Zoltán Zsolt Nagy, Matthias D. Becker, Gábor Márk Somfai

**Affiliations:** 1 Department of Ophthalmology, Stadtspital Zürich, 8063 Zurich, Switzerland; ferhat.turgut@stadtspital.ch (F.T.); tahm.spitznagel@stadtspital.ch (T.S.);; 2 Spross Research Institute, 8055 Zurich, Switzerland; 3 Gutblick Research, 8088 Pfäffikon, Switzerland; 4 Department of Ophthalmology, Semmelweis University, 1428 Budapest, Hungary; 5 Medical Dataway AG, 6300 Zug, Switzerland; 6 New York Eye and Ear Infirmary of Mt Sinai, Icahn School of Medicine, New York, NY 10029, USA; mddesmet1@mac.com; 7 MIOS sa/Helvetia Retina Associates, 1005 Lausanne, Switzerland; 8 Chargé de Recherche, Université Libre de Bruxelles, 1050 Brussels, Belgium; 9 Department of Ophthalmology, University of Heidelberg, 69117 Heidelberg, Germany

**Keywords:** real-time optical coherence tomography, epiretinal membrane peeling, intraoperative visualization, vitreoretinal surgery, surgical image processing, feature point alignment, robot-assisted surgery, surgical training

## Abstract

(1) Background: The process of epiretinal membrane peeling (MP) requires precise intraoperative visualization to achieve optimal surgical outcomes. This study investigates the integration of preoperative Optical Coherence Tomography (OCT) images into real-time surgical video feeds, providing a dynamic overlay that enhances the decision-making process during surgery. (2) Methods: Five MP surgeries were analyzed, where preoperative OCT images were first manually aligned with the initial frame of the surgical video by selecting five pairs of corresponding points. A homography transformation was then computed to overlay the OCT onto that first frame. Subsequently, for consecutive frames, feature point extraction (the Shi–Tomasi method) and optical flow computation (the Lucas–Kanade algorithm) were used to calculate frame-by-frame transformations, which were applied to the OCT image to maintain alignment in near real time. (3) Results: The method achieved a 92.7% success rate in optical flow detection and maintained an average processing speed of 7.56 frames per second (FPS), demonstrating the feasibility of near real-time application. (4) Conclusions: The developed approach facilitates enhanced intraoperative visualization, providing surgeons with easier retinal structure identification which results in more comprehensive data-driven decisions. By improving surgical precision while potentially reducing complications, this technique benefits both surgeons and patients. Furthermore, the integration of OCT overlays holds promise for advancing robot-assisted surgery and surgical training protocols. This pilot study establishes the feasibility of real-time OCT integration in MP and opens avenues for broader applications in vitreoretinal procedures.

## 1. Introduction

An epiretinal membrane (ERM) is a pathological fibrous layer that forms on the retinal surface, predominantly affecting the macular region [1]. This condition alters the normal architecture of the retinal layers and may lead to distorted vision (metamorphopsia), reduced visual acuity, and other visual impairments [2]. The pathophysiology of ERM involves an interplay between cellular proliferation, inflammatory responses, and the migration of myofibroblasts, glial cells, and retinal pigment epithelial cells onto the internal limiting membrane (ILM) [3,4]. As the membrane contracts, it exerts traction on the retinal layer bellow, causing macular distortion and structural changes [5].

ERM peeling (MP) surgery is a delicate procedure designed to restore retinal structure and function. During the procedure, the surgeon removes the fibrotic membrane from the macula with microforceps, often with the aid of dyes which improve visualization [6]. This surgery demands a high level of precision to prevent damage to the fragile retinal layers beneath, particularly as the ILM is often peeled as well to reduce the risk of recurrence [7,8,9]. Despite technical advancements, achieving optimal surgical outcomes remains challenging due to the microscopic scale and complexity of the affected structures [10].

Clear visualization of the retinal structures is crucial for the success of MP. Preoperative imaging, namely with Optical Coherence Tomography (OCT), has transformed diagnostics, providing high-resolution, cross-sectional views of the retina [11]. OCT allows for a detailed evaluation of the macular anatomy, membrane characteristics, and surgical planning. However, intraoperative visualization remains a far more complex challenge. While modern surgical microscopes, such as the ARTEVO 800 Digital Microscope (Carl Zeiss Meditec AG, Jena, Germany) and the Ngenuity 3D Visualization System (Alcon Laboratories Inc., Fort Worth, TX, USA), integrate OCT B-scans into the surgical field, the instruments themselves, such as forceps used for MP, may block the visual signal causing shadows that obscure or distort the visualization of the membrane at critical moments [12,13]. To overcome such limitations, new solutions are needed to enhance real-time visualization during surgery.

In this pilot study, we present a cost-effective and computationally efficient method for the integration of pre-operatively acquired OCT thickness maps into real-time surgical video overlays. Using classical image processing techniques, this new approach aligns OCT data with live surgical feeds, addressing the limitations of existing visualization systems. This method aims to enhance intraoperative visualization, increase surgical precision, and offer valuable insights for training and robot-assisted vitreoretinal procedures, all without the need for specialized intraoperative OCT devices.

## 2. Materials and Methods

This single-center, retrospective clinical study was conducted in the Department of Ophthalmology of the Stadtspital Zürich, Zürich, Switzerland, and included surgeries performed between January 2022 and December 2022. Approval for the study was obtained from the institutional ethics committee (IRB number 2023-01867). The study adhered to the Declaration of Helsinki and all federal and state laws of Switzerland. All patients provided a general consent for the use of non-personal data for scientific purposes. Inclusion criteria encompassed patients aged 18 years or older, diagnosed with an epiretinal membrane, and scheduled for vitrectomy with MP.

Preoperative OCT images by the Spectralis^®^ OCT (Heidelberg Engineering, Heidelberg, Germany) were acquired as part of the standard surgical planning process. The selection of surgical videos for this study was based on the following criteria: high-resolution (1024 × 576 WSVGA) recordings, minimal camera movement, shaking or obstructions, and complete peeling sequence to ensure comprehensive analysis. To minimize visual artifacts, only cases with optimal optical quality were included, such as surgeries performed on pseudophakic eyes or eyes without corneal opacities, reducing potential distortions that could impact the algorithm’s performance evaluation.

Surgical videos were segmented into individual frames for analysis. Each frame was manually assessed to ensure accurate alignment. Obscure or out-of-focus frames were excluded to maintain data consistency and reliability.

The computational pipeline was developed in Python 3.12.0 and tested on an Apple MacBook Pro (M1 Max, 10 cores, 32 GB RAM; Apple Inc., Cupertino, CA, USA). This setup provided adequate processing power for video alignment. Since Python is hardware-agnostic, the method can be adapted to various computing environments, including ARM-based edge devices (e.g., NVIDIA Jetson by NVIDIA Corporation, Santa Clara, CA, USA) and x86-based clinical workstations without significant modifications. This flexibility ensures broader applicability across a variety of computing environments without substantial code modifications.

**Figure 1:**
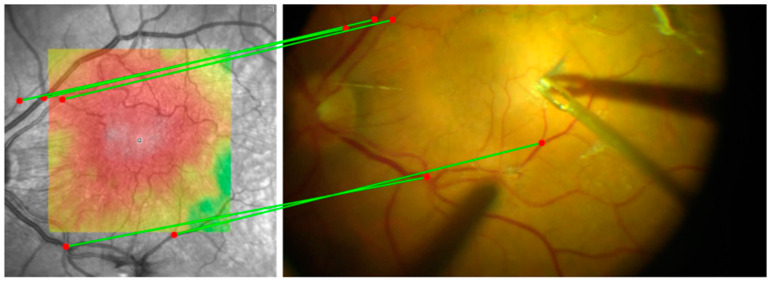
Manual selection of corresponding points for initial frame alignment. Five pairs of matching points (red markers) are shown on the OCT image (**left**) and the corresponding locations in the initial frame (**right**). These manually assigned points establish the reference homography for OCT-to-video alignment. The green lines illustrate the correspondence between the selected matching points in both images.

**Figure 2:**
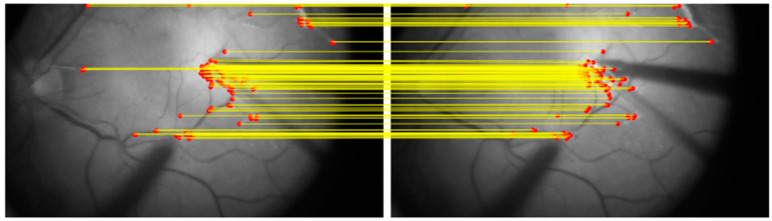
Illustration of frame-by-frame matching using Shi–Tomasi corner detection and Lucas–Kanade optical flow. Corners are detected in one frame (**left**) and tracked into the next frame (**right**). Colored lines represent the matching of corresponding corners across frames. The selected feature points, primarily concentrated near critical retinal landmarks such as vascular structures, highlight the targeted approach for ensuring precise alignment across consecutive video frames during surgery.

**Figure 3:**
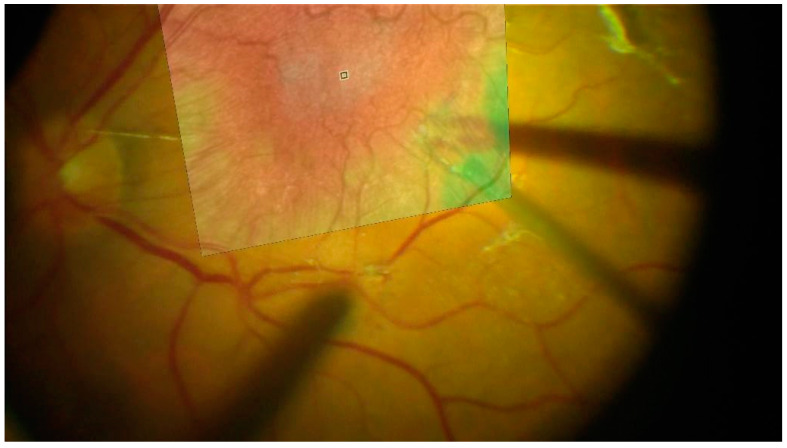
Overlay of the transformed OCT image onto a surgical video frame. The OCT thickness map (pseudocolor) remains aligned with the live surgical view as the instrument moves and the eye is repositioned.

To ensure precise and continuous alignment of the preoperative OCT image throughout the surgical video, the following stepwise approach is applied:

1. Manual Initialization: Five corresponding landmark points are manually selected on both the OCT image and the first frame of the surgical video. Each pair consists of a matching point in the OCT image and its corresponding point in the video frame (Figure 1). These points are used solely to align the OCT image with the initial video frame.
2. Homography Calculation: Using the manually selected key points, a homography transformation matrix is computed via the Direct Linear Transformation (DLT) algorithm in OpenCV [14]. This matrix warps the OCT image to match the first frame precisely.
3. Frame-to-Frame Transformation: After the initial alignment, a classical method is applied to calculate the transformation matrix between consecutive video frames. Specifically, corner features are detected in each frame using the Shi–Tomasi corner detection method [15], and the Lucas–Kanade optical flow algorithm [16] tracks these features across frames (Figure 2).
4. OCT Image Alignment Across Frames: The transformation matrix computed from the frame-to-frame alignment is then applied to the OCT image, ensuring that it remains aligned with every frame in the sequence.
5. Dynamic Adjustment: As the surgeon moves instruments or adjusts the viewing angle, the transformation matrix is continuously updated. This real-time recalibration maintains the accuracy of the OCT overlay throughout the procedure (Figure 3; Video S1).

## 3. Results

Surgical recordings (ARTEVO, Carl Zeiss Carl Zeiss Meditec AG, Jena, Germany) of five MPs were included.

The method demonstrated successful optical flow detection and feature point matching in 92.7 ± 4.2% of the analyzed consecutive 9068 frames (Table 1).

The algorithm, utilizing the Shi–Tomasi corner detection method and the Lucas–Kanade optical flow algorithm, showed a preference for selecting feature points near the vascular tree, which supported robust alignment. The videos achieved an average processing rate of 7.56 frames per second (FPS), highlighting the feasibility of a near real-time application.

## 4. Discussion

The results of this pilot study demonstrate the feasibility of integrating real-time OCT overlays into MP surgeries. The method’s high success rate of optical flow detection (92.7%) demonstrates strong reliability while using classical image-processing techniques in maintaining consistent frame correspondence, even without advanced machine learning or artificial intelligence algorithms. The algorithm’s preference for feature points near vascular structures further confirms its strategic selection process, contributing to robust alignment and ensuring that critical retinal regions remain accurately represented throughout the procedure.

MP is a complex vitreoretinal surgical procedure that requires meticulous precision and real-time decision-making. The advancement in imaging technologies, particularly OCT, has significantly improved preoperative diagnostics by providing high-resolution, cross-sectional views of the retina. Preoperative OCT allows surgeons to assess the macular structure and membrane thickness, and determine the optimal surgical approach [11,17]. Despite these advancements, intraoperative OCT guidance during MP has several limitations. These include shadow artifacts caused by surgical instruments, such as forceps, and the inability to visualize the forceps tip within the OCT image. Since preoperative imaging cannot capture dynamic intraoperative changes, surgeons must rely on static preoperative data, which may not accurately reflect real-time surgical conditions. Integrating real-time OCT overlays into the surgical feed could address these challenges by providing contextual information on membrane thickness and its proximity to the underlying retina. This enhancement would improve the precision of surgical maneuvers, particularly during the initiation of membrane peeling, potentially reducing surgical duration and ultimately benefiting patient outcomes.

One key advantage of this method is its adaptability with existing surgical video setups, which potentially reduces the barrier to adoption. Unlike more complex intraoperative OCT systems that require specialized hardware, this approach relies on computational tools that can be integrated into standard surgical equipment, making real-time OCT overlays more accessible for smaller clinics and research centers. Notably, this method requires only an off-the-shelf PC to function effectively. The current implementation was tested by using an Apple M1 Max CPU, which provided sufficient computational power for the algorithm, demonstrating its feasibility on commercially available hardware.

The average processing rate of 7.56 FPS, while sufficient for preliminary validation, is an area for improvement. Optimizing the algorithm and incorporating hardware acceleration could enhance the algorithm performance, ensuring a smoother, more natural experience for surgeons. This limitation arises from the balance between algorithmic demands and the capabilities of the hardware used. The current implementation utilized a commercially available, mid-range processor, as the method was designed to operate efficiently on standard PCs, making it accessible for smaller clinics. However, further optimization could allow the algorithm to fully leverage higher-performance hardware, increasing processing speed and scalability while maintaining compatibility with lower-end setups. Additionally, the algorithm does not yet utilize distributed computing or parallel processing, both of which could boost the processing speed. Integrating distributed computing approaches or optimizing the algorithm for GPU acceleration would likely improve performance, ensuring seamless real-time functionality in dynamic surgical environments.

Moreover, while classical image-processing methods have demonstrated robust performance, their integration with more advanced computer vision techniques, such as deep learning, could further enhance both the processing speed and accuracy. Studies in fundus imaging have shown that deep learning-based approaches achieve higher accuracy in retinal image alignment compared to our current method, highlighting the potential benefits of hybrid methodologies [18]. Additionally, to ensure data reliability, frames with significant obscuration or focus issues were excluded from the analysis. This underscores the need to achieve not only higher frame rates but also consistent frame quality for effective clinical application. Implementing these enhancements would improve the feasibility of real-time application, while ensuring the system meets the demands of dynamic surgical environments.

### 4.1. Impact of Real-Time OCT Overlays on Surgery and Training

The integration of real-time OCT overlays offers significant advantages for both surgeons and patients. For surgeons, the immediate visualization of OCT data within the surgical video enhances decision-making by providing real-time anatomical reference points. This capability allows for precise identification of retinal structures and better assessment of surgical progress, reducing the risk of complications and ensuring more accurate membrane removal.

This study serves as a technical proof of concept, demonstrating that a real-time OCT overlay is feasible using classical image-processing techniques. While the current implementation is not yet integrated into surgical microscopes, the findings suggest that such an approach could enhance intraoperative visualization, improve spatial awareness, and support surgical decision-making. Future developments would require collaboration with industry partners to integrate OCT overlays directly into surgical displays, potentially benefiting robotic-assisted surgery, surgical training programs, and real-time intraoperative guidance.

For patients, these advancements may lead to better surgical outcomes, shorter recovery times, fewer complications, and potentially fewer follow-up procedures. The combination of improved intraoperative precision and real-time feedback could elevate the overall standard of care, enhancing both safety and surgical efficacy in vitreoretinal procedures.

Additionally, this study highlights opportunities to enhance training protocols in vitreoretinal surgery. The real-time integration of OCT data can provide trainees with a more comprehensive understanding of retinal anatomy and surgical techniques, potentially shortening the learning curve and increasing surgical proficiency.

### 4.2. Limitations

This study is limited by several factors, most notably the small sample size of five surgical videos, which restricts the generalizability of the findings and prevents comprehensive statistical analysis. However, as a proof-of-concept, this study successfully validates the methodology and identifies key areas for further development. The selected cases provided sufficient data to evaluate the feasibility and performance of the proposed method while balancing computational and time constraints. This approach allows for focused testing of critical algorithmic capabilities, such as optical flow detection and OCT overlay alignment, under controlled conditions.

The small sample size underscores the need for future studies incorporating larger and more diverse datasets. Expanding the dataset to include videos from a broader range of surgical conditions, patient demographics, and optical environments will allow for more comprehensive statistical analyses and improve the generalizability of the findings. A larger-scale validation would not only enhance the reliability of the findings but also provide deeper insights into the method’s robustness and scalability across different clinical scenarios. Moving beyond this pilot study, large-scale validation is a crucial next step toward refining and integrating this technology into real-world surgical workflows.

While this proof-of-concept study demonstrates technical feasibility, its clinical applicability remains to be validated in real surgical environments. The small sample size limits statistical power, and the lack of direct intraoperative testing prevents assessment of real-time usability. While this proof-of-concept study confirms the feasibility of real-time OCT overlays, its clinical impact remains unverified. Future validation should focus on:

- Real-Time Usability Assessments: Conducting intraoperative trials where surgeons actively use the overlay system and provide structured feedback on its utility, accuracy, and impact on decision-making.
- Comparative Studies: Comparing surgical outcomes with and without OCT overlays to determine whether the technology improves precision, reduces complications, or decreases overall procedure time.
- Multicenter Validation: Expanding data collection across different surgical settings and patient populations to ensure that the system generalizes well beyond controlled experimental conditions.

By addressing these factors, future research can establish the clinical relevance of real-time OCT overlays and accelerate their integration into standard ophthalmic practice.

Beyond the sample size limitations, the processing rate of 7.56 FPS, while adequate for this pilot study, may not be sufficient for complex, real-time surgical environments. Hardware limitations and algorithmic processing delays present challenges that must be overcome to enable seamless integration in high-stakes surgical procedures. Additionally, the manual annotation of feature points in the initial frames adds an element of subjectivity, which could affect reproducibility. Automating this process and conducting further testing in live surgical settings would be necessary to fully realize the potential of this approach. Moreover, the method relies on manually adjusted parameters, requiring reconfiguration for each new video. This dependency on manual tuning not only limits scalability but also reduces its practicality in real-world applications. Developing automated parameter adjustment mechanisms, potentially leveraging machine learning techniques, would be essential to improve usability and adaptability, ensuring greater efficiency and broader clinical applicability.

## 5. Conclusions

This study demonstrates the successful and efficient overlay of OCT images onto MP surgery videos using classical algorithms. The real-time integration of OCT data could not only support surgical decision-making and the development of autonomous robot-assisted surgery but also has potential applications in surgical training [19,20]. Future studies should focus on improving frame processing speed through hardware acceleration, exploring hybrid deep-learning approaches for enhanced alignment, and validating the system in real-time clinical environments to optimize usability and efficacy.

## Figures and Tables

**Table 1:** Optical flow detection success rates and feature points near vascular structures. Points near vascular structures (%) were calculated by randomly extracting 100 frames from each video and visually inspecting them.

Video ID	Number of Frames (n)	Success Rate (%)	Points Near Vascular Structures (%)
Video 1	1033	91.5	83
Video 2	1524	84.4	67
Video 3	2398	92.9	84
Video 4	746	95.4	76
Video 5	3367	96.1	88
Total	9068	-	-
Mean ± Standard Deviation	1813.6 ± 957.9	92.7 ± 4.2	79.6 ± 7.4

## Data Availability

The datasets generated and/or analyzed during the current study are available from the corresponding author upon reasonable request.

## Supplementary Materials

The following supporting information can be downloaded at: https://www.mdpi.com/article/10.3390/bioengineering12030271/s1, Video S1: Real-Time Overlay of OCT Thickness Map onto Surgical Video.
